# Discriminative validity of Clinical High-Risk Status for Psychosis among help-seekers: a systematic review and meta-analysis

**DOI:** 10.1038/s41537-026-00785-4

**Published:** 2026-07-16

**Authors:** Andrea Raballo, Antonio Preti, Michele Poletti

**Affiliations:** 1 Chair of Psychiatry, Faculty of Biomedical Sciences, University of Southern Switzerland, Lugano, Switzerland; 2 Cantonal Sociopsychiatric Organisation, Mendrisio, Switzerland; 3 Department of Neuroscience, University of Turin, Turin, Italy; 4 Department of Mental Health and Pathological Addiction, Child and Adolescent Neuropsychiatry Service, Azienda Unità Sanitaria Locale-IRCCS di Reggio Emilia, Reggio Emilia, Italy

**Keywords:** Psychosis, Schizophrenia

## Abstract

The Clinical High-Risk for Psychosis (CHR-P) paradigm underpins early detection strategies and informs the DSM-5 construct of Attenuated Psychosis Syndrome (APS), yet its discriminative predictive validity relative to CHR-P–negative help-seekers has not been systematically quantified. We conducted a systematic review and meta-analysis of longitudinal studies comparing transition to psychosis among help-seeking individuals according to CHR-P status. PubMed/MEDLINE and the Cochrane Library were searched from inception to June 22, 2026. Eligible studies included help-seeking samples assessed with validated CHR-P instruments and reporting psychosis transition outcomes separately for CHR-P-positive and CHR-P-negative participants. Random-effects meta-analyses estimated transition prevalence and relative risks (RRs), while heterogeneity, publication bias, and moderators were examined. 14 studies including 4477 participants (2220 CHR-P–positive and 2257 CHR-P–negative) met inclusion criteria. Estimated transition rates were 14.7% [95% CI, 9.7–21.7%) among CHR-P-positive individuals and 1.6% (95% CI, 0.8–3.2%) among CHR-P-negative individuals. CHR-P status was associated with an almost ten-fold increased risk of transition to psychosis (RR, 9.8; 95% CI, 4.5–21.82), with no significant moderation by follow-up duration or sample characteristics. These findings demonstrate strong discriminative and predictive validity of CHR-P criteria among help-seekers, supporting attenuated psychotic symptoms as a clinically meaningful pre-psychotic phenotype. Results support consideration of APS in DSM-6 within a prognostically grounded, stepped-care framework rather than as a categorical disorder.

## Introduction

The Clinical High-Risk for Psychosis (CHR-P) paradigm has become a central framework for the early identification and prevention of psychotic disorders, grounded in the operationalization of subthreshold psychotic phenomena, functional decline, and genetic vulnerability^1^. Its construct and clinical validity rest on the assumption that attenuated psychotic symptoms represent a meaningful, preclinical phase along a psychosis continuum^2^, while its predictive validity is primarily evaluated through longitudinal transition to psychosis^2,3^.

Empirical evidence has consistently demonstrated elevated transition rates among individuals meeting CHR-P criteria compared to general population samples^2^; however, the literature has largely neglected systematic investigation of psychosis onset among help-seeking individuals who do not meet CHR-P thresholds. This asymmetry has limited the field’s capacity to assess the discriminative and prognostic boundaries of the construct, as predictive validity requires not only risk enrichment within CHR-P groups but also demonstrable risk differentiation from clinically similar, non-CHR-P help-seeking populations.

The key nosological question is whether attenuated symptoms meaningfully stratify prognosis among help-seeking individuals already enriched for distress. DSM-5 introduced, and DSM-5-TR kept Attenuated Psychosis Syndrome (APS) as a condition for further study, reflecting uncertainty about its clinical validity, diagnostic utility, and risk of premature medicalization. Towards DSM-6, evaluating construct and predictive validity through longitudinal comparisons of CHR-P-positive and CHR-P-negative help-seekers is essential. This approach clarifies whether current risk criteria identify a distinct pathway to psychosis or broader psychopathological vulnerability. This systematic review and meta-analysis examines longitudinal transition rates by CHR-P status to determine whether current thresholds provide meaningful prognostic separation.

## Methods

The following search engines were probed: PubMed/Medline and the Cochrane Library. The time interval was from inception up to June 22, 2026. A combination of the following key terms was applied: “ultra high risk” OR “clinical high risk” OR “basic symptoms” OR COGDIS OR COPER OR “SPI-A” AND psychosis AND (transition OR conversion). Search results were independently screened by two reviewers, each with more than ten years of experience in conducting systematic reviews, and verified by a third researcher with expertise in the field. Studies were considered eligible when they met the predefined inclusion criteria. Any disagreements regarding study eligibility or the identification of duplicate reports were resolved through discussion with a third senior reviewer until consensus was reached. The reference lists of all included articles were also examined for additional studies that may not have been captured by the database searches.

The following criteria were applied to retain an article in the study: the article was written in English; it included samples with people diagnosed CHR-P and considered CHR-P negative based on a validated diagnostic procedure; it reported numeric data about the samples (CHR-P positive and negative) and the outcome at a predefined follow-up time; it had transition to psychosis, as defined according to pre-specified criteria, as one of the outcomes.

Two experienced reviewers (MP and AP) independently extracted study data using a predefined data extraction form. The following information was collected: first author and year of publication, study location, diagnostic criteria and assessment instruments, criteria used to define transition to psychosis, sample size at baseline and follow-up, mean participant age, sex distribution, and duration of follow-up. This study adhered to the Preferred Reporting Items for Systematic Reviews and Meta-Analyses (PRISMA) guideline^4^.

### Statistical analyses

Statistical analyses were performed using IBM SPSS Statistics for Windows (Version 30.0) and R (Version 4.3.2; R Core Team, 2023). Meta-analytic procedures were conducted in R using the meta package^5^ and the metafor package^6^. Statistical significance was set at 2-sided α < 0.05, and we reported both point estimations and 95% confidence intervals (CI).

CHR-P operationalization overlaps with (but is not identical to) DSM-5 APS; the present analysis evaluated prognostic separation associated with CHR-P status in help-seeking populations, informing APS field evaluation rather than constituting direct DSM field testing.

Transition to psychosis (transition prevalence) was estimated using a random-intercept logistic regression model with logit transformation^7^. A random-effects model was adopted to account for the assumption that the underlying effect size may vary across studies owing to differences in study populations, settings, and methodologies. Under this framework, the pooled estimate reflects the average effect in the population of studies from which the included investigations may be considered a sample, allowing inference beyond the specific studies included in the meta-analysis^8^. However, when between-study heterogeneity is substantial, the pooled effect should be interpreted cautiously, as it may provide only a limited summary of effects that differ meaningfully across populations and contexts^9^.

Transition estimates were calculated according to the intention-to-treat principle, using the baseline sample size as the denominator. Participants who were lost to follow-up were not considered to have transitioned to psychosis, consistent with the outcome reporting of the original studies. Pairwise meta-analyses compared transition rates between help-seekers classified as CHR-P and those not classified as CHR-P. Relative risk (RR) with 95% confidence intervals (CIs) was calculated using the inverse variance method and the Mantel-Haenszel approach under random-effects models. Between- and within-studies variance were estimated with the τ2 statistics using the Empirical Bayes estimator^10^ and its 95% CI, which was calculated by using the Q-Profile method^6^ with Knapp and Hartung correction^11^.

Heterogeneity was assessed with Cochran’s Q and I² statistics^12^. A low *p*-value (i.e., *p* < 0.10) of the Q-statistic indicates that variation in the study-specific effect estimates is due to heterogeneity beyond that depending on sampling error^13^. The higher the I^2^, the greater the impact of the variance in true effects beyond that depending on sampling error^13^. According to an agreed rule-of-thumb, I^2^ values 0% to 40% might not be important; 30% to 60% may represent moderate heterogeneity; 50% to 90% may represent substantial heterogeneity^9^.

Funnel plot and the Egger’s regression test^14^ and the Begg’s test^15^ were applied to evaluate publication bias. Model adequacy was assessed with Galbraith’s radial plots^16^, with standardized residuals >2 indicating poor fit. Meta-regression was used to investigate the influence of study and sample characteristics on transition estimates. The examined moderators included sample size, follow-up duration, mean participant age, sex distribution, and dropout rate among CHR-P–positive participants.

In the presence of substantial heterogeneity, we planned a series of sensitivity analyses. First, we compared studies conducted in adult samples with those involving minors. Second, we repeated the analyses after excluding the study that identified high-risk individuals using latent class analysis. Third, we conducted an additional sensitivity analysis excluding studies with zero transition events. Finally, we compared samples defined by CHR-P criteria with those identified using basic symptom criteria. This distinction was considered relevant because samples identified using Comprehensive Assessment of At-Risk Mental States (CAARMS) or Structured Interview for Psychosis-Risk Syndromes (SIPS) criteria^17,18^ are generally regarded as more closely aligned with the proposed APS construct than samples identified solely through basic symptom criteria^19^ (e.g., SPI-A [Schizophrenia Proneness Instrument, Adult version], SPI-CY [Schizophrenia Proneness Instrument, Child and Youth version], COGDIS [Cognitive Disturbances], or COPER [Cognitive-Perceptive Basic Symptoms]). APS was largely derived from the attenuated positive symptom component of the CHR-P framework^20^. By contrast, basic symptom criteria originate from a distinct, although partially overlapping, approach to the identification of psychosis risk. Basic symptoms comprise subtle, self-experienced disturbances in cognitive, perceptual, linguistic, or affective processes that are typically recognized by the individual as abnormal. Unlike attenuated psychotic symptoms, these experiences arise from a different conceptual and theoretical tradition, namely the Bonn School model of psychosis vulnerability^21^.

## Results

The search yielded 2107 articles, of which 401 underwent full-text review; 14 studies^17,22–34^ satisfied the inclusion criteria (Figure S1). Table 1 provides a synthesis of their main characteristics. All studies compared cases meeting CHR-P criteria with non-CHR-P cases, except for one study^32^, which used latent class analysis to identify multiple subgroups, including one with a low probability of CHR-P symptoms (*n* = 523) and another characterized by a high probability of attenuated CHR-P positive symptoms and marked functional impairment (*n* = 97).

**Table 1:** Studies included in the meta-analysis, comparing longitudinal transition rates in CHR-P positive and CHR-P negative help-seekers.

First Author, Year	CHR-P instrument	Follow up Months	baseline CHR-P negative sample *n* =	Follow up sample *n* =	Raw transitions *n* =	Baseline CHR-P positive sample *n* =	Follow up sample *n* =	Raw transition *n* =	Mean age (SD) CHR-P Neg Years =	Gender CHR-P neg %F	Mean age (SD) CHR-P Pos Years =	Gender CHR-P pos %F
Klosterkotter, 2001	BSABS	116	132	50	2	253	110	77	30,62 (10,33)	50	28,1(9,75)	46,4
Yung, 2005	CAARMS	6	107	107	1	43	43	5	21,1	62,5	19,1	49
Woods, 2009	SIPS	30	198	135	3	377	303	89	16,1	36,4	18,2	62,1
Nelson, 2011	CAARMS	6	494	494	0	434	434	72	21,7 (3.4)	42,5	median 18	59
Simon, 2012	SIPS	24	49	18	0	73	56	10	21,6 (5)	20	20,4 (5,2)	39,7
Lindgren, 2014	SIPS	12	107	92	1	54	51	2	16,7 (0,85)	77,6	16,7 (0,85)	81,5
Schultze-Lutter, 2014	SIPS	24	52	52	7	311	194	74	24,9	39,2	24.87 (6)	37
Manninen, 2014	SIPS	60	45	40	1	7	7	1	15-18		15-18	
Francesconi, 2017	CAARMS	36	71	61	4	71	62	4	24,2 (3,6)	51,4	24,5 (3,4)	42,2
Mensi, 2021	CAARMS	60	102	91	1	110	103	21	15,3	65,7	15,5	62,7
Haining, 2024	CAARMS	36	51	51	0	144	116	14	22,88 (4.74)	66,7	21,63 (4,14)	66,7
Stüble, 2024	SIPS	48	523	523	5	97	97	38	16,23 (4,6)	49,9	19,9 (5.2)	51,1
Tykka, 2025	SIPS	15	49	49	0	63	63	3	19,49 (3,19)	50,8	19,89 (3,02)	53,1
Cabrera, 2026	SIPS/SPI-CY	24	277	173	1	183	85	12	14,26 (2,54)	62,3	15,30 (2,07)	54,5

*BSABS* Bonn Scale for the Assessment of Basic Symptoms, *CAARMS* Comprehensive Assessment of At-Risk Mental State; *CHR-P* Clinical High-Risk for Psychosis, *SD* Standard Deviation, *SIPS* Structured Interview for Prodromal Symptoms, *SPI-CY* Schizophrenia Proneness Instrument, Child & Youth version.

CHR-P status and psychosis transition were determined using the SIPS in 8 studies, the CAARMS in 5 studies, and the Bonn Scale for the Assessment of Basic Symptoms (BSABS) in 1 study. Furthermore, one study employing the SIPS also assessed Basic Symptoms using the SPI-CY, documenting a substantial prevalence of Basic Symptoms among participants.

The duration of follow-up ranged from 6 to 116 months (mean, 36 months; SD = 30). Dropout ranged from 0 to 143 participants in the CHR-P-positive group and from 0 to 104 participants in the CHR-P-negative group. Overall, 496 participants (22.3%) in the CHR-P-positive group and 321 participants (14.2%) in the CHR-P-negative group were lost to follow-up. Across samples, mean dropout rates were 17% (SD, 20%) and 17% (SD, 23%), respectively.

A total of 2220 help-seekers who met CHR-P criteria were included, of whom 422 transitioned to psychosis during follow-up. Among the 2257 help-seekers who did not meet CHR-P criteria, 26 transitioned. Across studies, the mean sample size was 159 (SD = 134) for the CHR-P-positive group and 161 (SD = 161) for the CHR-P-negative group.

Age was reported for all samples, and sex distribution was reported for 13 samples. The mean age was 19.9 years (SD = 4) in the CHR-P-positive group and 20.0 years (SD = 5) in the CHR-P-negative group. Women accounted for 54% (SD = 12%) and 47% (SD = 16%) of participants, respectively, indicating a modest excess of women in the CHR-P–positive group. Among the eight studies reporting this information, the proportion of participants with attenuated psychotic symptoms ranged from 61% to 100%, reaching ≥96% in six. All included studies originated from Western countries, except for one conducted in India^33^; overall, the studies were of good methodological quality. (Table S1).

**Fig. 1:**
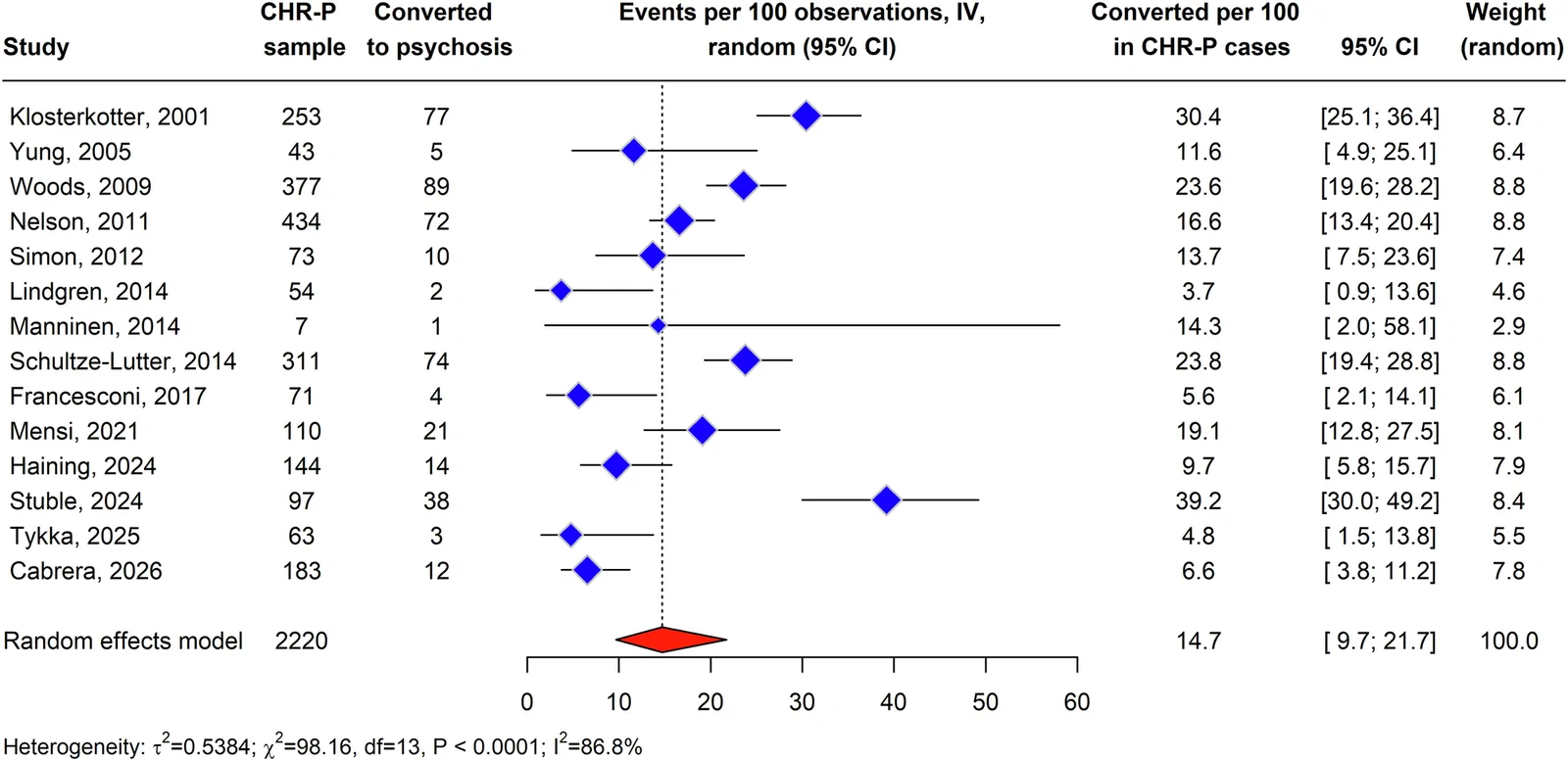
Forest plot of the estimated prevalence of transition to psychosis among CHR-P–positive help-seeking individuals. Random-effects meta-analysis of transition rates across included studies. Squares represent study-specific estimates (size proportional to study weight), horizontal lines indicate 95% confidence intervals, and the diamond represents the pooled estimate.

**Fig. 2:**
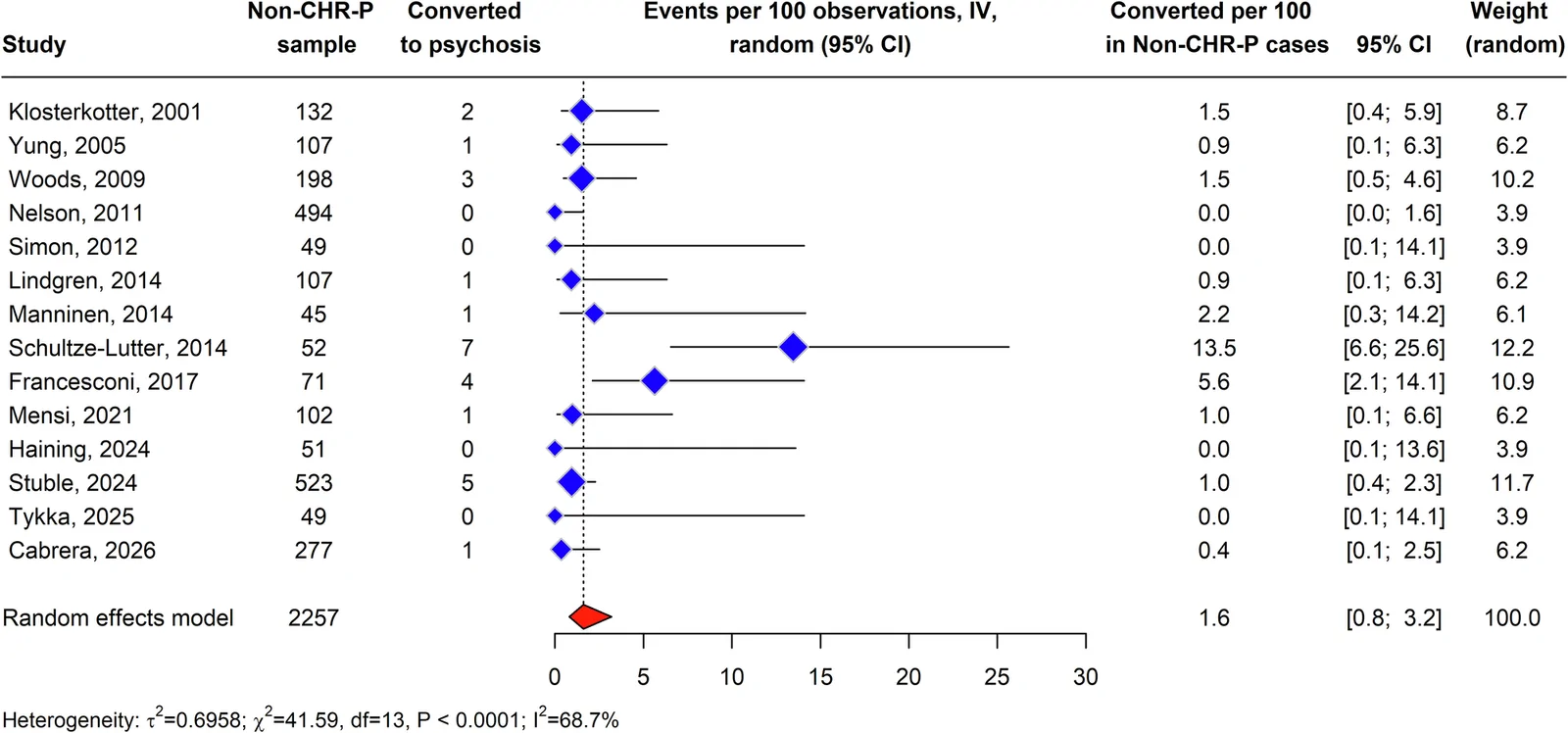
Forest plot of the estimated prevalence of transition to psychosis among CHR-P–negative help-seeking individuals. Random-effects meta-analysis of transition rates across included studies. Squares represent study-specific estimates (size proportional to study weight), horizontal lines indicate 95% confidence intervals, and the diamond represents the pooled estimate.

**Fig. 3:**
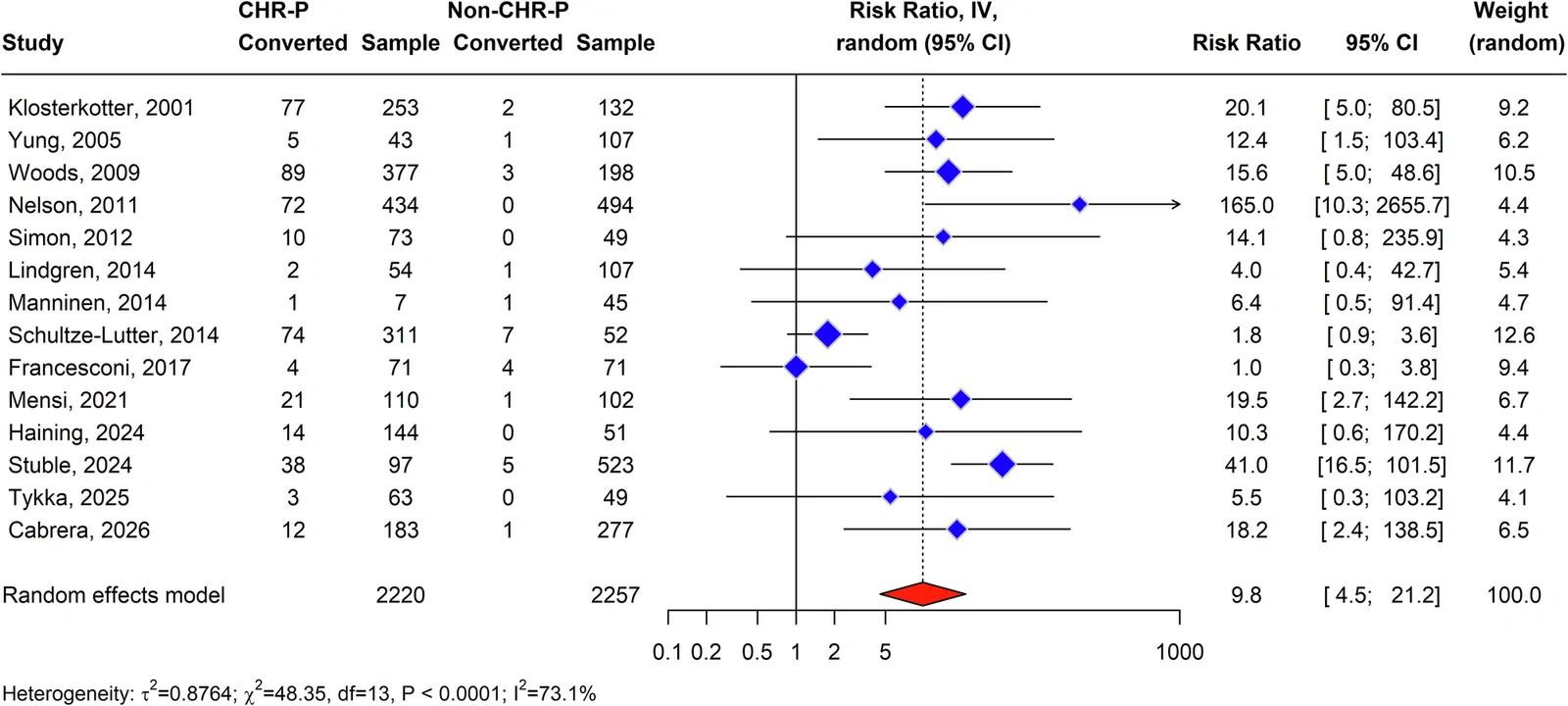
Forest plot of the relative Risk of transition to psychosis in Help-Seeking individuals with CHR-P–Positive vs CHR-P-Negative status. Random-effects model (inverse-variance, Mantel–Haenszel). Squares indicate study-specific estimates (size proportional to weight); horizontal lines, 95% CIs; diamond, pooled estimate.

The random-effects model estimated a transition rate of 14.7% [95% CI, 9.7%-21.7% (Fig. 1)] in the CHR-P-positive group and 1.6% [0.8%-3.2% (Fig. 2)] in the CHR-P-negative group, with substantial heterogeneity observed in both analyses. CHR-P–positive cases were nearly ten times more likely to transition to psychosis than CHR-P-negative cases (RR = 9.8; 4.5–21.2; Fig. 3).

**Fig. 4:**
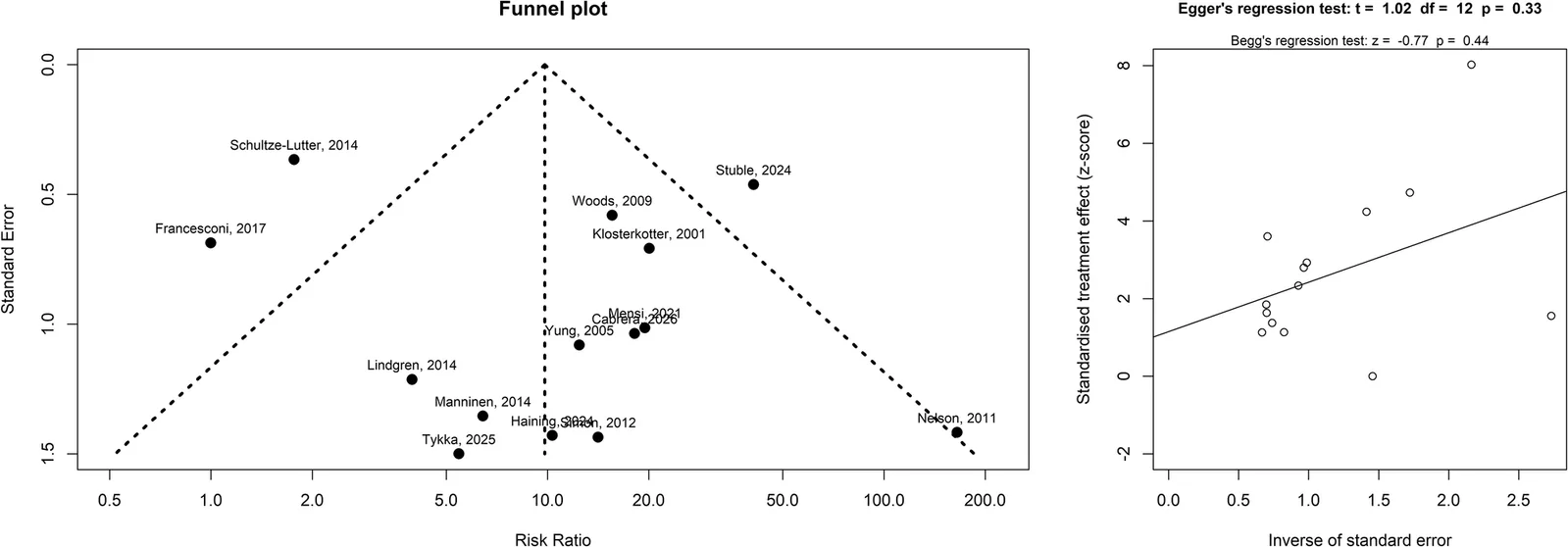
Funnel plot and small-study effect tests for the Relative Risk meta-analysis. Visual inspection and Egger/Begg tests showed no signal detected.

The funnel plot appeared reasonably symmetric, and Egger and Begg regression tests did not indicate evidence of publication bias (Fig. 4).

**Fig. 5:**
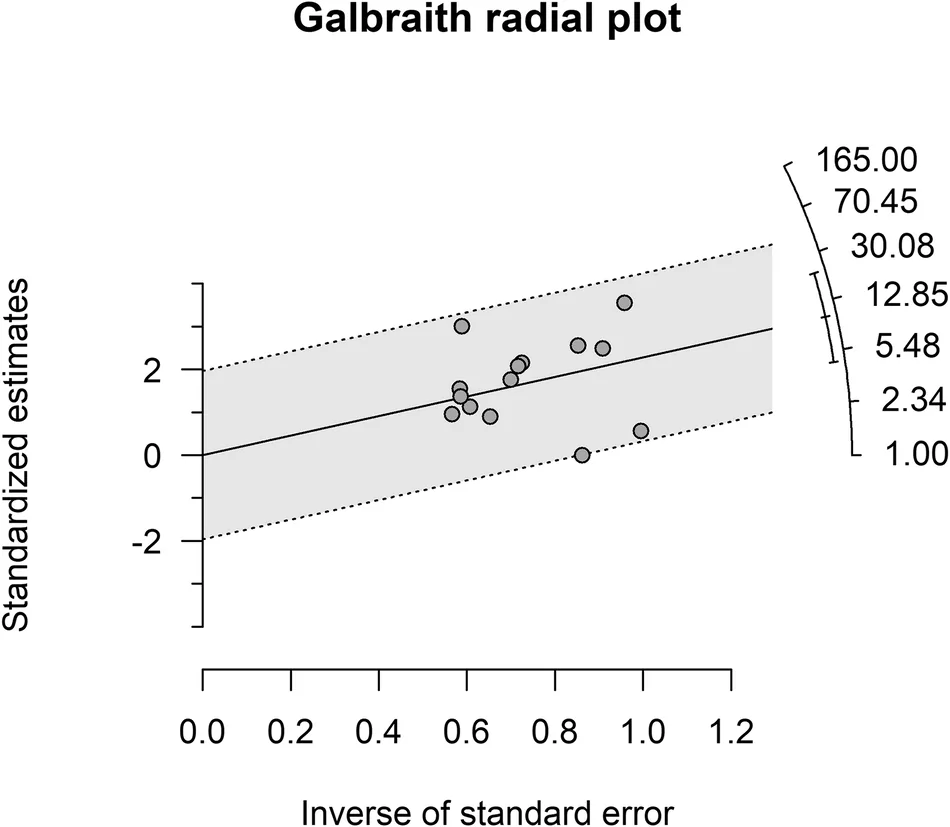
Galbraith (radial) plot of the relative risk of transition to psychosis among help-seeking individuals with CHR-P–positive versus CHR-P–negative status. The plot is based on intention-to-treat data and was used to assess model fit and identify potential outlying studies. Points lying beyond the reference limits indicate studies contributing disproportionately to heterogeneity.

The radial plot indicated a good fit of the (random effects) model (Fig. 5).

Sample size in CHR-P positive (β = 0.002; 95% CI, –0.004 to 0.008; *p* = 0.48), follow-up duration (β = 0.008; 95% CI, –0.019 to 0.035; *p* = 0.53), age (β = –0.14; 95% CI, –0.32 to 0.05; *p* = 0.14), sex (β = 0.04; 95% CI, –0.03 to 0.11; *p* = 0.24), and dropout rate (β = -0.009; 95% CI, –0.05 to 0.03; *p* = 0.64) were not associated with RR estimates.

The sample assessed with the BSABS showed a higher RR (20; 95% CI, 1.7 to 236) compared with samples assessed using the CAARMS (9.3; 0.9 to 95.3) or the SIPS (9.2; 3.5 to 24). However, these differences were not statistically significant (Q = 0.35; df = 2; *p* = 0.84).

In the sensitivity analysis excluding cases of the study based on latent class analysis classification^32^, the risk of transition to psychosis was confirmed higher in CHR-P-positive individuals than in CHR-P–negative individuals (RR = 7.9, 95% CI 3.6–17.5). Statistical heterogeneity remained substantial (I² = 62.8%, 95% CI 32.2 to 79.5%; χ² = 32.2, df = 12, *p* = 0.0013).

After excluding four studies with zero transition events in the CHR-P–negative group, CHR-P–positive individuals remained at markedly increased risk of psychosis transition (RR = 8.5, 95% CI 3.4–21.5). Between-study heterogeneity remained substantial (I² = 79.2%, 95% CI 62.3–88.5%; χ² = 43.2, df = 9, *p* < 0.0001).

The pooled risk ratio was similar in samples of minors and young adults (RR = 11.1 vs 10.1). Heterogeneity appeared lower among studies of minors, although this finding should be interpreted cautiously given the limited number of studies (*n* = 4; Fig. S2).

In the two studies assessing basic symptoms, the relative risk for psychosis transition was higher than that observed in studies applying CHR-P criteria alone (RR = 19.5 vs 9.8). Heterogeneity was lower in this subgroup, although this finding should be interpreted cautiously given the very limited number of studies (*n* = 2; Fig. S3).

## Discussion

This meta-analysis shows that CHR-P status produces a substantial post-test risk shift in help-seeking populations, with an absolute transition difference of about 13 percentage points and a near ten-fold relative risk. This level of prognostic separation indicates that CHR-P criteria function as a clinically meaningful risk stratifier within the referral context in which they are applied, directly supporting the discriminative validity of current operational thresholds. Although absolute transition rates varied across studies, relative risk estimates remained remarkably consistent, a distinction that is clinically important. Absolute risk is influenced by contextual factors such as referral pathways, pre-test risk enrichment, service configuration, treatment exposure, and duration of monitoring. Consequently, the baseline probability of transition may differ substantially across settings. By contrast, the discriminative function of CHR-P criteria reflects their ability to stratify risk within a given help-seeking population. The present findings indicate that, despite variation in absolute risk, individuals meeting CHR-P criteria consistently showed a markedly higher risk of psychosis onset than clinically comparable CHR-P-negative help-seekers. This pattern suggests that CHR-P criteria retain prognostic value across different service contexts even when absolute transition rates fluctuate. Aggregated data further indicate high sensitivity and negative predictive value, supporting CHR-P as a staging and triage construct rather than a categorical diagnosis.

Although substantial between-study heterogeneity was observed, neither meta-regression nor sensitivity analyses materially altered the pooled estimates or explained a meaningful proportion of variance. This suggests that the prognostic separation associated with CHR-P status is relatively robust across different study designs and populations, while also indicating that heterogeneity may arise from unmeasured factors such as referral pathways, service configuration, treatment exposure, ascertainment procedures, or differences in baseline risk enrichment.

Conceptualizing CHR-P as a staging and triage construct implies that its primary clinical function is not to establish a categorical diagnosis but to guide the intensity and type of intervention. Among help-seekers, individuals meeting CHR-P criteria may warrant enhanced monitoring, psychoeducation, family involvement, and evidence-based psychological interventions while avoiding premature diagnostic labeling and unnecessary exposure to intensive treatments^35^. Within this framework, CHR-P criteria support the allocation of preventive resources according to estimated risk rather than defining a discrete disease entity.

These findings are consistent with clinical staging models, in which psychosis risk syndromes are conceptualized as intermediate stages along a continuum of psychopathological development rather than as discrete disorders^36^. Within this framework, stepped-care approaches permit intervention intensity to be matched to the individual’s level of risk, clinical needs, and functional impairment, thereby maximizing preventive benefit while minimizing overtreatment^37^.

The numerically higher RR observed in studies assessing Basic Symptoms may reflect the identification of a subgroup within the broader CHR-P spectrum characterized by particularly elevated psychosis liability. Given the substantial, although incomplete, overlap between Basic Symptoms and CHR-P criteria, these findings are more consistent with complementary rather than competing models of psychosis-risk detection^37^.

While CHR-P criteria are not identical to DSM-5 APS, attenuated psychosis symptoms account for the large majority (approximately 85%) of CHR-P cases in international cohorts^38^, with BLIPS and genetic risk-deterioration subgroups comprising smaller proportions. Accordingly, the observed prognostic separation largely reflects the attenuated positive symptom domain central to APS. Although not a formal DSM field validation, these findings provide indirect but substantial support for the representativeness and clinical relevance of APS in real-world help-seeking populations.

The persistence of a low but measurable transition rate among CHR-P-negative help-seekers indicates that psychosis liability extends beyond current thresholds, which function as risk stratification boundaries rather than natural disease demarcations. CHR-P criteria appear to identify a late, high-risk segment of a broader developmental vulnerability continuum, consistent with dimensional models of early psychopathology.

These findings provide empirical support for considering APS in DSM-6 as a prognostically anchored risk syndrome within stepped-care staging frameworks rather than as a standalone categorical disorder, with clinical utility grounded in calibrated risk enrichment and longitudinal monitoring.

These findings should be interpreted in light of the limited number of studies, compared with those conducted on CHR-P populations, that have longitudinally investigated help-seeking individuals who did not meet CHR-P criteria at baseline. This aspect, which reflects the overall quality and scope of the included and analyzed studies, suggests that the implications and conclusions of the present review are based on only a small subset of studies adopting the CHR-P paradigm.

Limitations include between-study heterogeneity, variable follow-up, reliance on transition outcomes, heterogeneous CHR-P negative comparison groups, and limited power to detect small-study effects. Future work should integrate functional and transdiagnostic trajectories beyond transition alone.

## Supplementary information

Supplementary Materials

## Data Availability

This systematic review was based on data from previously published articles; no original data were collected. All data generated or analyzed during this study are included in this article or the supplement. Further inquiries can be directed to the corresponding author.
