# Supplementary material for: Discriminative validity of Clinical High-Risk Status for Psychosis among help-seekers: a systematic review and meta-analysis
Source: Schizophrenia (Heidelb). 2026 Jul 16;12(1):86. doi: 10.1038/s41537-026-00785-4 (PMC13745538; doi:10.1038/s41537-026-00785-4)

Supplementary Materials

Figure S1.

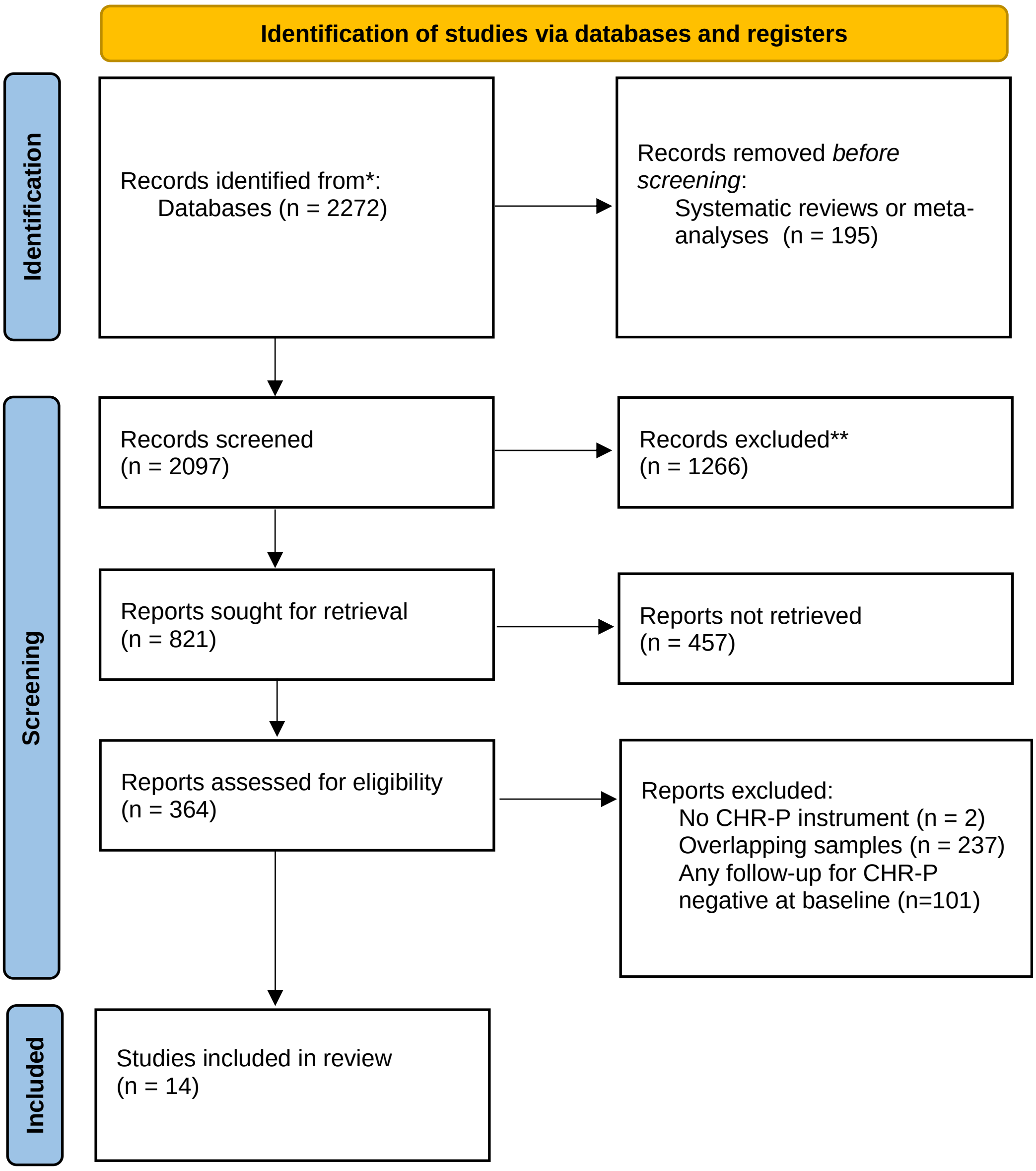

*Consider, if feasible to do so, reporting the number of records identified from each database or register searched (rather than the total number across all databases/registers).

**If automation tools were used, indicate how many records were excluded by a human and how many were excluded by automation tools.

Source: Page MJ, et al. BMJ 2021;372:n71. doi: 10.1136/bmj.n71.

This work is licensed under CC BY 4.0. To view a copy of this license, visit https://creativecommons.org/licenses/by/4.0/

Supplementary Materials

Table S1 Quality rating of studies included in the Meta-analysis according to the Newcastle-Ottawa Assessment Form for Cohort Studies.

Study	Selection				Comparability	Outcome			AHRQ Standard
First Author, year	Item 1	Item 2	Item 3	Item 4	Item 1	Item 1	Item 2	Item 3	
Klosterkotter, 2001	*	*	*	*	*	*	*	*	Good
Yung, 2005	*	*	*	*	*	*	*	*	Good
Woods, 2009	*	*	*	*	*	*	*	*	Good
Nelson, 2011	*	*	*	*	*		*	*	Good
Simon, 2012	*	*	*	*	*	*	*	*	Good
Lindgren, 2014	*	*	*	*	*	*	*	*	Good
Manninen, 2014		*	*	*	*	*	*	*	Good
Francesconi, 2017	*	*	*	*	*	*	*	*	Good
Mensi, 2021	*	*	*	*	*	*	*	*	Good
Haining, 2024	*	*	*	*	*	*	*	*	Good
Stuble, 2024	*	*	*	*	*	*	*	*	Good
Tykka, 2025	*	*	*	*	*	*	*	*	Good
Cabrera., 2026	*	*	*	*	*	*	*	*	Good

Note: Newcastle-Ottawa Quality Assessment Form for Cohort Studies

Selection items

1) Representativeness of the exposed cohort

2) Selection of the non-exposed cohort

3) Ascertainment of exposure

4) Demonstration that outcome of interest was not present at start of study

Comparability Items

1) Comparability of cohorts on the basis of the design or analysis controlled for confounders

Outcome items

1) Assessment of outcome

2) Was follow-up long enough for outcomes to occur

3) Adequacy of follow-up of cohorts

Thresholds for converting the Newcastle-Ottawa scales to AHRQ standards (good, fair, and poor):

Good quality: 3 or 4 stars in selection domain AND 1 or 2 stars in comparability domain AND 2 or 3 stars in outcome/exposure domain

Fair quality: 2 stars in selection domain AND 1 or 2 stars in comparability domain AND 2 or 3 stars in outcome/exposure domain

Supplementary Materials

Poor quality: 0 or 1 star in selection domain OR 0 stars in comparability domain OR 0 or 1 stars in outcome/exposure domain

Figure S2. Forest plot of the relative risk of transition to psychosis among help-seeking individuals with CHR-P–positive versus CHR-P–negative status: subgroup analysis comparing studies of minors (children and adolescents) and young adults - Random-effects meta-analysis using the inverse-variance Mantel–Haenszel method. Squares represent study-specific relative risk estimates (with sizes proportional to study weights), horizontal lines indicate 95% confidence intervals, and diamonds represent pooled estimates.

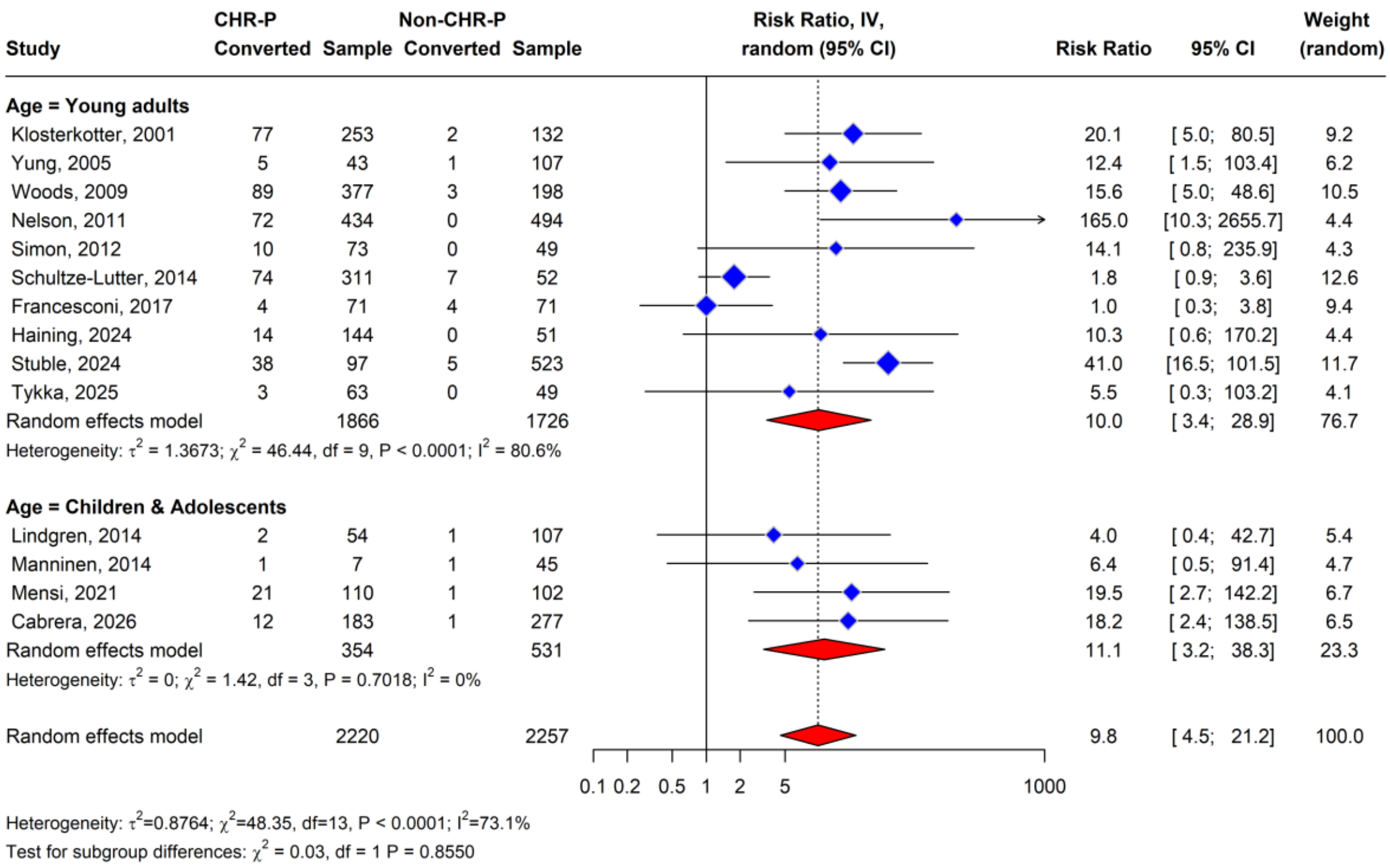

Supplementary Materials

Figure S3. Forest plot of the relative risk of transition to psychosis among help-seeking individuals with CHR-P–positive versus CHR-P–negative status: subgroup analysis comparing studies incorporating Basic Symptoms assessment and studies based exclusively on CHR-P criteria - Random-effects meta-analysis using the inverse-variance Mantel–Haenszel method. Squares represent study-specific relative risk estimates (with sizes proportional to study weights), horizontal lines indicate 95% confidence intervals, and diamonds represent pooled estimates.

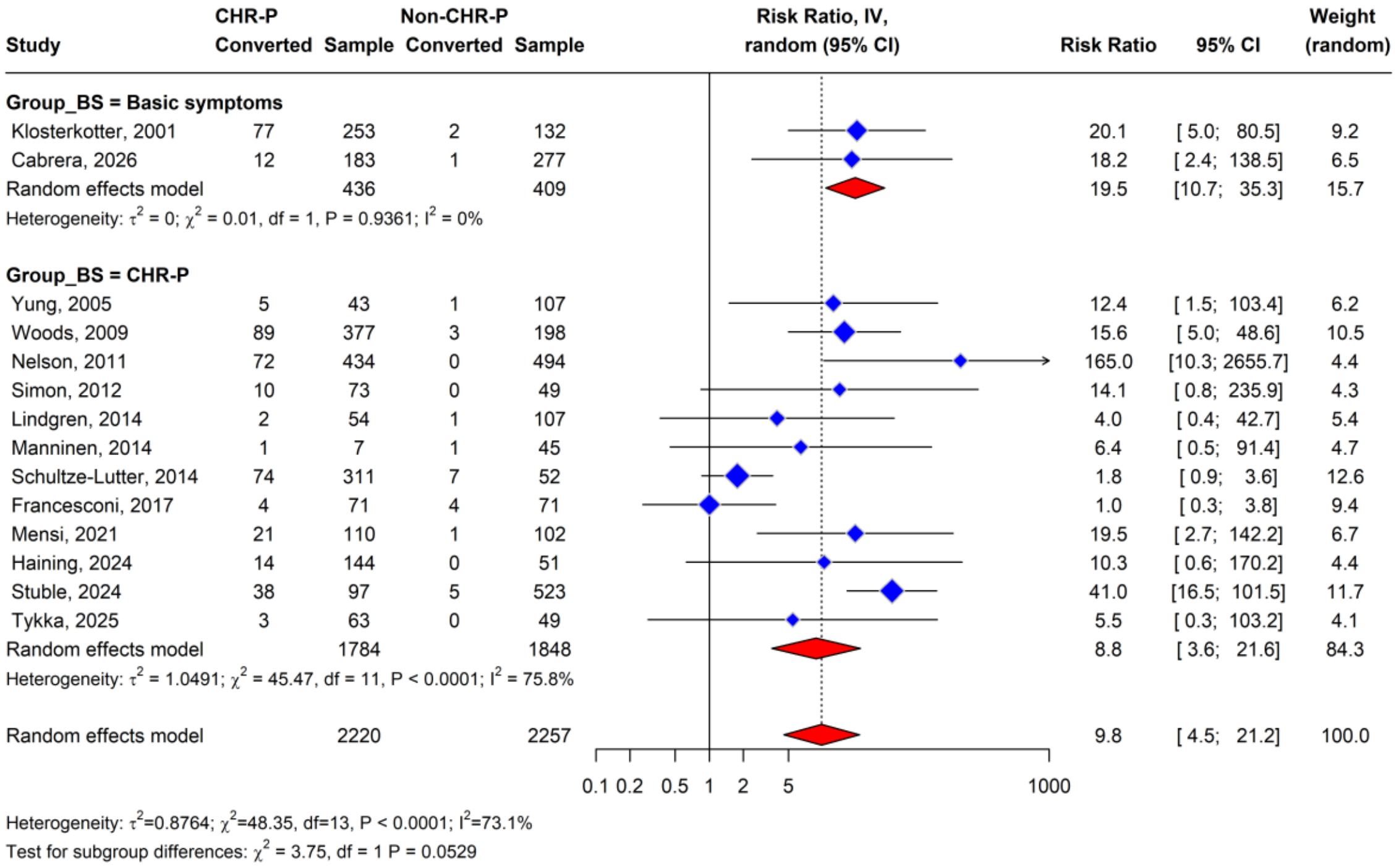

Supplement: Supplementary file 1 [file 41537_2026_785_MOESM1_ESM.pdf]
